# Development and Content Validation of an Instrument to Measure Medication Self-Management in Older Adults

**DOI:** 10.3390/pharmacy9020078

**Published:** 2021-04-11

**Authors:** Tejal Patel, Aidan McDougall, Jessica Ivo, Jillian Carducci, Sarah Pritchard, Feng Chang, Sadaf Faisal, Catherine Lee

**Affiliations:** 1 School of Pharmacy, University of Waterloo, 10 Victorial St S., Kitchener, ON N2G 1C5, Canada; jarivo@uwaterloo.ca (J.I.); feng.chang@uwaterloo.ca (F.C.); sadaf.faisal@uwaterloo.ca (S.F.); 2 Centre for Family Medicine Family Health Team, 10 Victoria St S., Kitchener, ON N2G 1C5, Canada; ahmcdougall@uwaterloo.ca (A.M.); jillian.carducci@family-medicine.ca (J.C.); sarah.pritchard@family-medicine.ca (S.P.); catherine.lee@uwaterloo.ca (C.L.); 3 Schlegel—University of Waterloo Research Institute of Aging, 250 Laurelwood Drive, Waterloo, ON N2J 0E2, Canada

**Keywords:** medication adherence, medication management, aged, elderly, validation study

## Abstract

Background: For older adults, the capacity to self-manage medications may be limited by several factors. However, currently available tools do not permit a comprehensive assessment of such limitations. The Domain Specific Limitation in Medication Management Capacity (DSL-MMC) was developed to address this need. This study aimed to establish the face and content validity of the DSL-MMC. Methods: The DSL-MMC tool consisted of 4 domains and 12 sub-domains with 42 items including: 1. physical abilities (vision, dexterity, hearing); 2. cognition (comprehension, memory, executive functioning); 3. medication regimen complexity (dosing regimen, non-oral administration, polypharmacy); and 4. access/caregiver (prescription refill, new prescription, caregiver). Pharmacists assessed each item for relevance, importance, readability, understandability, and representation. Items with content validity index (CVI) scores of <0.80 for relevance were examined for revision or removal. Results: Twelve pharmacists participated in the study. CVI scores for relevance and importance of domains were 1.0; of the sub-domains, two were below 0.80. Among the 42 items, 35 (83%) and 30 (71%) maintained CVI scores above 0.80 for relevance and importance, respectively. Five items were removed, three were merged and seven were modified due to low CVI scores and/or feedback. Conclusion: The DSL-MMC has been validated for content.

## 1. Introduction

Canada’s population has been aging steadily as noted by the annual increase in mean age since record keeping began in 1971. Indeed, in the last year, the largest growth rate in the population has been among centenarians at 10.4% as compared to the growth rate in overall population at 1.1%. As of July 2020, 18.0% of Canada’s population is composed of those aged 65 years and older. By 2025, it is projected that 1 in 5 Canadians will be an older adult, and by 2059, 25% of the population will be composed of seniors [1]. 

These remarkable statistics speak to the need for pharmacists to gain expertise in the healthcare needs of an aging population, which differs from that of the general population. Aging is generally accompanied by multimorbidity, that is the coexistence of two or more medical conditions [2]. For example, the prevalence of multimorbidity in those aged less than 65 years is 50.3% compared to 62% among the 65–74-year-old, 75.7% among the 75–84-year-old population and 81.5% among those aged 85 years and older [2]. As expected, highly prevalent conditions includes hypertension, hyperlipidemia, ischemic heart disease, diabetes, arthritis, heart failure, depression, chronic kidney disease, osteoporosis, Alzheimer’s disease, chronic obstructive pulmonary disease, atrial fibrillation, cancer, asthma and stroke [2]. Since most chronic medical conditions are treated with medications, it is not surprising that in Canada, older adults are prescribed 6.9 different medications, on average, and that almost 25% are prescribed 10 or more medications [3]. Unfortunately, use of multiple medications increases the complexity of medication taking, a contributor to treatment-related factors driving non-adherence [4,5,6,7,8]. The economic cost of non-adherence on the healthcare systems is tremendous. In the United States, the adjusted total cost of medication non-adherence ranges from US $949 to $52,341 annually across all disease states [9]. Despite the significant cost and effort to address non-adherence, rates have not improved over the past 15 years [10]. One reason may be the lack of matching the intervention to patient specific determinants for medication non-adherence; the two are hardly ever matched in clinical trials. For example, Haynes et al. found only 13% of studies in their systematic review examined the reasons for non-adherence at baseline [11,12].

Medication adherence, defined as the extent to which a patient acts in concordance with the dose and dosing interval of a medication prescribed by a healthcare professional [5], is strongly dependent upon a patient’s medication management capacity, or the “cognitive and functional ability to self-administer a medication regimen, as it has been prescribed [13]”. Medication taking is a complex process that requires both physical and cognitive functioning. For example, medication administration requires the ability to read and understand instructions for dosing as provided on the prescription label. Furthermore, patients have to be able to identify the right container or package, open the container or blister packaging, and remove the appropriate number of tablets or capsules. They have to be able to hold the medication unit between their fingertips or in the palm of their hand before swallowing it [14]. For non-oral medications, patients should be able to follow more complex instructions; for example, in applying and removing patches or actuating inhalers and following the appropriate process for inhaling medications. 

Unfortunately, aging and accompanying multimorbidity is associated with declining physical and cognitive health which results in functional impairment and impacts the ability to self-administer medications [15]. For example, in one of the earliest studies conducted to examine medication taking ability, 78.3% of the participants (mean age 81.8 +/− 6.2 years) were unable to open a container or split a tablet [16]. A more recent study demonstrated that among their younger population (mean age 74 years), 28.4% experienced some problem with opening the medication packaging [17]. Other studies have also demonstrated the impact of vision acuity and hearing impairment on unintentional medication errors [18,19]. Besides age-related physical limitations in strength, vision and hearing, cognitive decline has also been associated with diminishing medication management capacity. In the study by Beckman et al., 23.9%, 31.5% and 38.4% of those aged 77–79 years, 80–84 years, and 85 years and older, respectively, did not understand dosing instructions [17]. Several other studies have also demonstrated the significant relationship between declining medication management capacity and impaired cognition [20,21,22,23,24]. 

The decline in an older adult’s ability to self-manage medications and increase in risk of medication errors, adverse drug events, placement in assisted living and hospitalization are well recognized in published literature [25,26,27,28]. Therefore, several instruments have been developed to examine a patient’s functional and cognitive capacity to manage medications, using the patient’s own medications or simulated medication regimen and tasks [29,30,31,32]. These instruments enable clinicians to examine both physical and cognitive skills in their older adults. However, many vary in their validity and reliability and none of these instruments are gold standard measures [29,32]. Furthermore, there is significant variability in the comprehensiveness of their assessments, in both the physical and cognitive skills [29,32,33]. For example, while 96% of the instruments assess skill in opening medication packaging, only 18% examine the ability to split a tablet. When examining cognitive skills, most instruments assessed reading standard medication labels (75%) but very few examined whether participants knew how many repeats were remaining (2.3%) [33]. For example, the Drug Regimen Unassisted Grading Scale (DRUGS) [20], the most used instrument in clinical research, requires patients to identify each of their medications appropriately, open the appropriate medication containers, dispense the correct number of pills and report on the appropriate timing of the doses. It does not, however, require an explicit examination of cognition, medication regimen complexity or the domains of access and caregiver support on the impact of medication management. It does permit an examination of a person’s physical functioning through an assessment of identifying and opening containers. Similarly, the Medication Management Instrument for Deficiencies in the Elderly (MedMaIDE) [34] does not explicitly assess vision, and memory but enables a clinician to determine whether a patient knows about their medications, knows how to take their medications and knows how to get their medications. We, therefore, developed the Domain Specific Limitation in Medication Management Capacity (DSL-MMC) Tool (see Supplemental Files) to comprehensively assess four domains and 12 sub-domains that may impact medication management capacity in older adults. The objective of this study was to examine the face and content validity of the DSL-MMC.

## 2. Materials and Methods

### 2.1. Design

To examine the face and content validity of the DSL-MMC tool, we conducted a prospective study with pharmacists in a 2-stage process as recommended by Lynn et al. [35]. We initially developed the tool, then invited clinicians to provide quantitative and qualitative feedback on the relevance, importance, readability, understandability and comprehensiveness of the tool, which was then used to further revise the instrument. 

### 2.2. Ethical Review

This study was reviewed by and received approval from the University of Waterloo Clinical Research Ethics Committee, (#23150)

### 2.3. Development of the Tool

The objective of the DSL-MMC is to measure the impact of domain specific limitations on the medication management capacity of older adults. We developed the tool by reviewing the published literature pertaining to both factors that impact, and tools that assess medication management capacity. We identified 42 items that would permit a comprehensive examination of the limitations older adults may face when managing their medications. These 42 items were grouped into 12 sub-domains, which were then grouped together into four domains. The four domains included “physical”, “cognitive”, “medication regimen complexity” and “access and caregiver”. 

Sub-domains grouped within the domain of “physical limitations” included “vision”, “hearing” and “dexterity” and the sub-domains, “memory”, “executive function” and “comprehension” were grouped into the domain of “cognition”. The sub-domains of “dosing regimen”, “non-oral administration” and “polypharmacy” were grouped in the domain of “medication regimen complexity” while the sub-domains of “new prescription”, “prescription refill” and “caregiver” were grouped together in the domain of “access and caregiver”.

Of the 42 items, 4 were question-based and 38 were performance-based items designed to examine medication management tasks. Examples of question-based items included “Do you have any difficulties hearing or do you have any hearing impairments?” and “Does anyone (family member, friend, other person) help you prepare or administer your medications?” while performance-based items included asking patients to read names of medications on prescription vials or demonstrate how an individual may return pills to the prescription vials. 

### 2.4. Sample

Healthcare professionals who either prescribed, reviewed, dispensed or assisted patients with medication management tasks were eligible to participate in this study. Purposive sampling technique, through professional networks, was used to recruit a sample of at least 10 participants. A minimum sample size of 3–10 participants is recommended for the purpose of examining content validation index [35,36]. Content experts who were involved in the development of the tool (TP, JC, SP) were excluded from the testing. 

### 2.5. Face and Content Validation

Content validity has been defined as “the degree to which a sample of items, taken together, constitute an adequate operational definition of a construct.” [36] Content validity is determined by examining the relevance of scale content by experts. Therefore, pharmacist participants—the experts—were provided with a paper document which contained the DSL-MMC, the purpose of the DSL-MMC, objective of the study, definitions of the terms used for both domains and sub-domains and instructions to examine the relevance, importance, readability and understandability of each of the 42 items through the use of paper-based surveys. Relevance and importance were assessed on a 4-point Likert scale, while readability and understandability were assessed via dichotomous “yes/no” questions. In addition to the items, participants were also asked to assess the relevance and importance of each sub-domain and domain. Participants also examined whether representation of domain and sub-domains was comprehensive, and whether there were any potential excluded or redundant items. Finally, participants were also invited to provide open-ended feedback. Participants completed their reviews and provided their feedback independently. 

### 2.6. Data Analysis

The percent agreement and content validity index (CVI) for each of the 42 items, 12 sub-domains and 4 domains was calculated utilizing Microsoft Office Excel 365 (version 2008 Build 13127.21348 Click-to-Run). Percent agreement refers to the proportion of participants who rated each item within each of the four options on the Likert Scale while the item—CVI (I-CVI) score was determined by the following formula [36]:

$$I-CVI=\;\frac{\left(n\;experts\;who\;score\;3\right)+\left(n\;experts\;who\;score\;4\right)}{\left(total\;n\;of\;experts\right)}$$

The “content valid” responses include those which were graded as 3 or 4 on the 4-point Likert scale where 3 and 4 corresponds to “important” or “relevant” or “absolutely important” or “absolutely relevant,” respectively. 

To examine the content validity index for the scale (S-CVI), we performed the following calculation [36]:

$$S-CVI=\frac{\left(sum\;of\;all\;I-CVI\;scores\right)}{\left(total\;n\;of\;scored\;items\right)}$$

Scores on readability and understandability were also examined for each item. Items with a CVI score of less than 0.80 for relevance were removed. Items with a CVI value of less than 0.80 for importance were examined further for removal or editing if respondents indicated low understandability and readability scores and provided feedback indicating as such in the open feedback section. 

## 3. Results

Twelve pharmacists provided consent to participate in the study (see Table 1).

Content validity index scores for relevance and importance of all the domains were 1.00 (see Table 2). The CVI scores for relevance of sub-domains ranged from 0.64 to 1.00 while that of importance of sub-domains ranged from 0.55 to 1.00. The CVI scores for two sub-domains were below 0.80: the CVI scores for both relevance (0.64) and importance (0.55) of the sub-domain of “hearing” and the CVI score for importance (0.73) for the sub-domain, “new prescription”. 

Among the individual items, 83% (35/42) and 71% (30/42) maintained relevance and importance CVI scores above 0.80, respectively. Ultimately, four items were removed due to having relevance CVI scores below 0.80 and one was removed due to a low understandability score. Three items were merged into one and seven items were revised due to feedback received on low importance, and to improve readability or understandability (see Table 3).

The removed items included the following: (1) separating individual medication units; (2) returning a tablet to a medication container; (3) calculate the duration of a medication; (4) preparing a medication while under a time constraint or while being timed and; (5) identifying the number of remaining refills. Three were felt to be redundant and merged based on feedback received from participants. The items in question were: (1) identify or differentiate a medication by color, (2) identify or differentiate a medication by shape, and (3) identify or differentiate the medication container (e.g., pill capsule). These items were merged into the following: (1) identify or differentiate the medication unit (e.g., identifying or differentiating the medication unit by shape or color) and specify how it was identified or differentiated. 

Of the items revised, six were adjusted to include examples to aid in readability and understandability. One item was below the CVI threshold of 0.80, but the said item had low readability and as such, this item was revised to improve its readability (see Supplemental Files).

The S-CVI for the DSL-MMC was 0.90.

The final version of DSL-MMC contains 35 items organized under the same four domains and 12 sub-domains.

## 4. Discussion

This study establishes the face and content validity for a new comprehensive instrument designed to assess all the domains that can impact medication management capacity in an older adult. All domains and majority of sub-domains included in this tool were deemed relevant and important with CVI scores of above 0.80. Sub-domains rated highly for relevance and importance included vision, dexterity, comprehension, executive function, dosing regimen, non-oral administration, and prescription refill. High CVI scores in these domains and sub-domains indicates that there is inter-rater consensus among content experts that these factors are important to consider when assessing an older adult’s ability to self-medicate.

Two sub-domains, “Hearing” (relevance 0.64, importance 0.55) and “New Prescription” (relevance 0.91, importance 0.73), had CVI values which scored lower than other sub-domains, indicating that pharmacist content experts did not consider these factors to be as relevant or important to consider when assessing an older adults’ ability to self-medicate. This was a particularly surprising finding as hearing impairments have been demonstrated to be negatively associated with therapeutic adherence. In one particular study, individuals with hearing impairment had twice the odds of non-adherence when compared to those without a hearing impairment [19]. Written feedback provided with regard to this sub-domain indicated that participants felt hearing could be assessed informally, through the use of indirect questions rather than a specific sub-domain dedicated to hearing. For instance, participants indicated rather than asking “Do you have difficulties hearing?”, hearing could be assessed by seeing if the patient has difficulty responding to other prompts within the interaction. However, hearing loss can be mild and progressive [37], resulting in a deceptive assumption of no hearing loss on the part of the clinician based on a conversation. For example, a cross-sectional study among primary care physicians and secondary care providers demonstrated 66.5% felt confident in their capacity to communicate with hearing impaired individuals even though only 13% of the physicians and 3.5% of the secondary care providers had attended formal training [38]. Furthermore, 10% of respondents indicated that medication errors had resulted from the miscommunication due to hearing impairment in their older adult patient population [38]. Screening for hearing loss with a single question such as, “Do you think you have hearing loss?” has a median positive likelihood ratio of 3.0 and a negative likelihood ratio of 0.40, indicating that single question screening may be useful for identification of potential hearing loss [37]. Additionally, since two of the three items in the sub-domain of “hearing” had CVI scores of >0.8 for relevancy we did not remove the subdomain of “hearing” and we edited to improve the readability of the item with a CVI score of less than 0.8 responding to a question in the “hearing” sub-domain.

In terms of the sub-domain, “new prescription”, the low CVI score (0.73) for importance may be due to its similarity to the sub-domain of “prescription refill”. Since the sub-domain of prescription refill appeared prior to new prescription, the perceived similarity between these two sub-domains by participants may have impacted the importance scoring for the latter domain. Although there are similarities, the sub-domain of “new prescription” evaluates the ability of a patient to recognize if there are no refills remaining and if so, understand the process of receiving a new prescription. Since this process of receiving a new prescription can differ from receiving a medication refill, it is important to assess these two subdomains separately rather than together. Furthermore, since the low CVI score was for the importance and not relevance, we did not remove these items from the DSL-MMC tool.

With the aging of the Canadian population [1], and increasing use of medications to address chronic conditions for longer periods of time [3], it is imperative that we provide community healthcare providers with the tools to examine an older adult’s capacity to self-manage medications. Indeed, pharmacists are in a key position to monitor adherence through their dispensing records and target non-adherence by selecting interventions based on patient reported barriers and facilitators [7,39]. Since a multitude of factors [5,6,7,8] can limit one’s ability to appropriately manage his or her medications, the use of a tool that comprehensively assesses numerous sub-domains related to medication management ability is necessary for identification of all potential contributors for medication mismanagement. The DSL-MMC will enable clinicians to assess the domain-specific limitations in medication management capacity among older adults and as such, determine appropriate limitation specific solutions to improve adherence to medications. However, before the DSL-MMC can be used in clinical practice, it requires further testing for inter-rater and test–retest reliability as well as predictive validity. Finally, the feasibility of implementing this tool in clinical practice needs to be examined before full scale use of this tool can be recommended. We also developed a standardized protocol for the administration of the tool in clinical practice. This is to ensure that further testing of the instrument is reproducible [29]. Finally, the content of this tool has been validated for use by pharmacists and must not be utilized as a self-report measure by older adults until it is validated for such use by older adults.

## 5. Limitations

One limitation of this study is the unbalanced representation of the sample, with all participants being pharmacists with a high percentage of them being female. Additionally, although we obtained qualitative feedback, additional measures of acquiring feedback, for example, using one-to-one interview or focus group methodologies, may have provided additional information with which to further improve the DSL-MMC. It would also have provided an avenue for clarification of some of the comments provided.

## 6. Conclusions

Older adults may face several challenges with the self-management of medications, including various physical, and cognitive limitations as well as increasing complexity in medication regimens. The items captured in the instrument, Domain Specific Limitations in Medication Management Capacity, permits a comprehensive assessment of the different factors that impact self-management of medications in older adults as demonstrated by an S-CVI of 0.90.

## Figures and Tables

**Table 1:** Pharmacist Demographic Information.

Demographic Variable	N = 12
**Gender (n, %)**	
Female	10 (83%)
Male	2 (17%)
**Years of Practice (years)**	
Mean	13.55
SD	11.08
Median	10
Range	1–36

**Table 2:** Pharmacist Ratings of Domains and Sub-Domains of a 42-Item Scale: Percent Agreement and Context Validity Index.

Domains and Sub-Domains	Relevance PA (%)				Relevance CVI	Importance PA (%)				Importance CVI
	NR	LR	R	AR		NI	LI	I	AI	
*Physical Abilities*	0	0	8	92	1.00	0	0	8	92	1.00
Vision	0	0	17	83	1.00	0	0	0	100	1.00
Dexterity	0	0	25	75	1.00	0	0	8	92	1.00
Hearing	9	36	36	18	0.64	0	36	36	27	0.55
*Cognition*	0	0	0	100	1.00	0	0	0	100	1.00
Comprehension	0	0	0	100	1.00	0	0	0	100	1.00
Memory	9	0	18	73	0.91	0	9	18	73	0.91
Executive Functioning	0	0	42	58	1.00	0	0	33	67	1.00
*Medication Regimen Complexity*	0	0	50	50	1.00	0	0	42	58	1.00
Dosing Regimen	0	8	33	58	1.00	0	0	42	58	0.92
Non-oral Administration	0	0	33	67	1.00	0	0	42	58	1.00
Polypharmacy	0	8	25	67	0.92	0	8	25	67	0.92
*Access and Caregiver*	0	0	42	58	1.00	0	0	33	67	1.00
Prescription Refill	0	0	67	33	1.00	0	0	67	33	1.00
New Prescription	9	18	36	36	0.91	9	0	45	45	0.73
Caregiver	0	0	42	58	0.92	8	0	33	58	1.00

PA = Percent Agreement (defined as the proportion of participants who picked that value); CVI = Context validity index; NR = Not Relevant; LR = Of Little Relevance; R = Relevant; AR = Absolutely Relevant; NI = Not Important; LI = Of Little Importance; I = Important; AI = Absolutely Important.

**Table 3:** Pharmacist Ratings of Items in a 42-Item Scale.

Domain	Item #	Importance PA				Importance CVI		Relevance PA				Relevance CVI		RD (%)	U (%)	Outcome
		NI	LI	I	AI			NR	LR	R	AR					
Vision	1	0	25	8	67	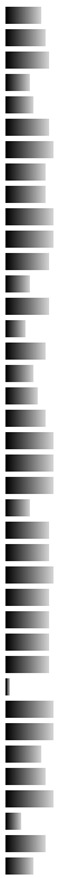 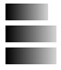	0.75	0	0	33	67	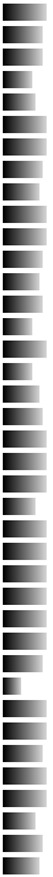 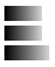	1.00	92	91	Revised
	2	0	17	17	67		0.83	0	8	17	75		0.92	67	47	Merged
	3	0	8	50	42		0.92	0	8	33	58		0.92	100	92	No Change
	4	8	42	25	25		0.50	0	33	33	33		0.67	100	100	Merged
	5	8	33	33	25		0.58	0	25	33	42		0.75	100	100	Merged
	6	0	8	25	67		0.92	0	0	33	67		1.00	100	92	No Change
Dexterity	7	0	0	17	83		1.00	0	0	8	92		1.00	100	100	No Change
	8	0	17	17	67		0.83	0	8	25	67		0.92	100	92	No Change
	9	0	17	25	58		0.83	0	17	17	67		0.83	75	58	Revised
	10	0	0	33	67		1.00	0	0	25	75		1.00	100	100	No Change
	11	0	0	25	75		1.00	0	0	8	92		1.00	100	100	No Change
	12	0	8	67	25		0.92	0	8	58	33		0.92	82	45	Removed
	13	0	50	25	25		0.50	0	17	50	33		0.83	100	100	No Change
	14	0	8	25	67		0.92	0	8	8	83		0.92	100	100	No Change
	15	0	58	42	0		0.42	0	33	58	8		0.67	100	100	Removed
	16	0	17	58	25		0.83	0	0	50	50		1.00	100	100	No Change
Hearing	17	8	33	33	25		0.58	0	33	17	50		0.67	58	83	Revised
	18	17	17	42	25		0.67	8	8	50	33		0.83	100	100	No Change
	19	0	17	33	50		0.83	0	8	33	58		0.92	100	100	No Change
Comp	20	0	0	0	100		1.00	0	0	0	100		1.00	100	100	No Change
	21	0	0	25	75		1.00	0	0	17	83		1.00	100	100	No Change
M	22	0	0	17	83		1.00	0	0	25	75		1.00	92	92	No Change
Exec Func	23	0	50	42	8		0.50	0	25	67	8		0.75	83	83	Removed
	24	0	8	58	33		0.92	0	0	67	33		1.00	100	100	No Change
	25	0	8	33	58		0.92	0	0	33	67		1.00	91	82	No Change
	26	0	0	25	75		1.00	0	0	17	83		1.00	100	90	No Change
Dosing Regimen	27	0	8	42	50		0.92	0	0	42	58		1.00	92	83	Revised
	28	0	8	25	67		0.92	0	0	33	67		1.00	83	83	Revised
	29	0	8	50	42		0.92	0	0	33	67		1.00	83	83	No Change
	30	0	8	33	58		0.92	0	8	25	67		0.92	100	92	Revised
	31	25	67	8	0		0.08	8	50	42	0		0.42	100	92	Removed
Non-Oral	32	0	0	42	58		1.00	0	0	33	67		1.00	100	83	No Change
	33	0	0	25	75		1.00	0	0	17	83		1.00	100	100	No Change
	34	0	25	33	42		0.75	0	8	50	42		0.92	100	100	No Change
Po	35	0	17	25	58		0.83	0	0	42	58		1.00	83	75	Revised
Rx Refill	36	0	0	42	58		1.00	0	0	42	58		1.00	100	100	No Change
	37	17	50	25	8		0.33	0	25	67	8		0.75	100	100	Removed
	38	0	17	25	58		0.83	0	8	17	75		0.92	100	100	No Change
New Rx	39	8	33	42	17		0.58	8	8	58	25		0.83	100	92	No Change
	40	0	17	42	42		0.83	0	17	25	58		0.83	83	92	No Change
CG	41	0	0	25	75		1.00	8	8	17	67		0.83	100	100	No Change
	42	0	0	33	67		1.00	0	0	36	64		1.00	100	92	No Change

PA = Percent Agreement; CVI = Context validity index; RD = Readable; U = Understandable; NR = Not Relevant; LR = Of Little Relevance; R = Relevant; AR = Absolutely Relevant; NI = Not Important; LI = Of Little Importance; I = Important; AI = Absolutely Important; Comp = Comprehension; M = Memory; Exec Func = Executive Functioning; Non-Oral = Non-Oral Administration; Po = Polypharmacy; Rx Refill = Prescription Refill; New Rx = New Prescription; CG = Caregiver

## Data Availability

The data from this study may be available from the corresponding author after a reasonable request and approval from the institutional review board.

## Supplementary Materials

The following are available online at https://www.mdpi.com/article/10.3390/pharmacy9020078/s1.
