# Supplementary material for: Development and Content Validation of an Instrument to Measure Medication Self-Management in Older Adults
Source: Pharmacy (Basel). 2021 Apr 11;9(2):78. doi: 10.3390/pharmacy9020078 (PMC8167785; doi:10.3390/pharmacy9020078)
Supplement: Supplementary file 1 [file pharmacy-09-00078-s001.pdf]

Supplemental File 1:

Domain Specific Limitation in Medication Management Capacity (DSL-MMC) Instrument

**DOMAIN: PHYSICAL ABILITIES**	
When assessing the impact of ***vision, dexterity*** and ***hearing*** on the capacity to manage medications, is an older adult able to:	
**Vision**	A. identify or differentiate the outer packaging of a medication (e.g. medication box)?
	B. identify or differentiate the medication container (e.g. pill capsule)?
	C. read instructions out loud? (e.g. reading the label of a pill bottle, blister pack, inhaler, etc.)
	D. differentiate medications by colour?
	E. differentiate medications by shape?
	F. differentiate strengths of the medication? (e.g. a 1 mg pill compared with a 5 mg pill)
**Dexterity**	A. open the outer medication packaging (e.g. medication box, plastic wrapper)?
	B. to break a tamper proof seal or security seal to gain access to a medication?
	C. open a medication container (e.g. pill capsule)?
	D. remove medication from a medication container? Note: this does not include blister packages
	E. remove medication from a blister package?
	F. separate individual medication units?
	G. split tablets?
	H. pick up or hold a tablet?
	I. return a tablet to a medication container?
	J. close a medication container?
**Hearing**	A. voice hearing difficulties or hearing impairments?
	B. utilize a hearing aid, if needed
	C. reiterate, interpret or respond to questions that were stated out loud?
**DOMAIN: COGNITION**	
When assessing the impact of ***comprehension (Comp), memory (Mem)*** and ***executive functioning (EF)*** on the capacity to manage medications, is an older adult able to:	
**Comp**	A. apply their understanding after reading a medication label?
	B. apply their understanding after hearing about a medication regimen?
**Mem**	A. remember and recall something throughout the interaction?
**EF**	A. calculate the duration of a medication?
	B. plan a medication dose in advance?
	C. identify over or under administration?
	D. go through the steps involved in preparing and administering medication without being prompted between steps?

COPYRIGHT © 2021 EMAP Research Program

DOMAIN: MEDICATION REGIMEN COMPLEXITY		
When assessing the impact of ***dosing regimen, non-oral administration (NOA)*** and ***polypharmacy (PolyRx)*** on the capacity to manage medications, is an older adult able to:		
**Dosing Regimen**	A.	administer multiple (> once daily) daily doses of the same medication?
	B.	administer multiple strengths of the same medication at different dosing times during a period of time?
	C.	adjust a medication dose based on titration or tapering schedules?
	D.	Carry out a medication regimen where there are special instructions?
	E.	Prepare and administer medication under a time constraint or while being timed
**NOA**	A.	Prepare non-oral medication, if applicable?
	B.	Administer non-oral medication, if applicable?
	C.	Dispose of non-oral medication after use, if applicable?
**Poly Rx**	A.	Simultaneously carry out five or more medication regimens (e.g. assess if they are on five or more medication regimens or create a fictitious regimen for them to follow)?

DOMAIN: ACCESS & CAREGIVER		
When assessing the impact of ***prescription refills (Rx Refll), new prescription (New RX)*** and ***caregiver (CG)*** on the capacity to manage medications, is an older adult able to:		
**Rx Refill**	A.	Identify the name of the medication that needs to be refilled?
	B.	Identify the number of remaining refills?
	C.	Identify whom to call to order a refill?
**New Rx**	A.	Identify when a new prescription is needed?
	B.	Identify whom to contact to attain a new prescription?
**CG**	A.	Identify if a caregiver aids in the medication preparation or administration process?

COPYRIGHT © 2021 EMAP Research Program

Supplemental File 2:

Summary of Revisions

Item Number	Original Wording	Revised Wording
1	Identify of differentiate the outer packaging of a medication (e.g. medication box)	Identify or differentiate the outer packaging of a medication (e.g. medication box, or pill vial/bottle)
2	Identify or differentiate the medication container (e.g. pill capsule)	Identify or differentiate the medication unit (e.g. identifying or differentiating the medication unit by shape or colour) and specify how it was identified or differentiated
9	Open a medication container (e.g. pill capsule)	Open a medication container (e.g. pill vial/bottle)
17	Voice hearing difficulties or hearing impairments	Express hearing difficulties or hearing impairments
27	Administer multiple (> once daily) daily doses of the same medication. *NOTE: this can be assessed by creating a fictitious regimen or by asking if the participant is on this type of regimen*	Administer multiple (> once daily) daily doses of the same medication. *NOTE: use the following fictitious regimen: Phenytoin, 100 mg, take 1 capsule in the morning, take 2 capsules in the evening by mouth*
28	Administer multiple strengths of the same medication at different dosing times during a period of time. *NOTE: this can be assessed by creating a fictitious regimen or by asking if the participant is on this type of regimen*	Administer multiple strengths of the same medication at different dosing times during a period of time. *NOTE: use the following fictitious regimen: Warfarin, 2 mg, take 1 tablet daily by mouth on Monday, Wednesday and Friday; and Warfarin, 3 mg, take 1 tablet daily by mouth on Tuesday, Thursday, Saturday and Sunday*
30	Carry out a medication regimen where there are special instructions. *NOTE: this can be assessed by creating a factious regimen or by asking if the participant is on this type of regimen*	Carry out a medication regimen where there are special instructions. *NOTE: use the following fictious regimen: Levothyroxine (Eltroxin), 50 mg, take 1 tablet daily by mouth, take with food*
35	Simultaneously carry out five or more medication regimens (e.g. assess if they are on five or more medication regimens or create a fictitious regimen for them to follow)	Simultaneously carry out five or more medication regimens (e.g. assess if they are able to manage 5 or more medications or use the following regimen: Phenytoin, 100 mg, take 1 capsule in the morning, take 2 capsules in the evening by mouth; Warfarin, 2 mg, take 1 tablet daily by mouth on Monday, Wednesday and Friday; Warfarin, 3 mg, take 1 tablet daily by mouth on Tuesday, Thursday, Saturday and Sunday; Propranolol, 20 mg, take half a tablet (10mg) for the first two days then take 1 tablet (20mg) daily by mouth; Pantoprazole, 20 mg, take 1 tablet twice a day by mouth, Vitamin D3
